# Point-of-Care Fluorescence Biosensing System for Rapid Multi-Allergen Screening †

**DOI:** 10.3390/s24113280

**Published:** 2024-05-21

**Authors:** Silvia Demuru, Hui Chai-Gao, Yevhen Shynkarenko, Nicola Hermann, Patricia-Daiana Boia, Peter Cristofolini, Bradley Petkus, Silvia Generelli, Samantha Paoletti, Stefano Cattaneo, Loïc Burr

**Affiliations:** Swiss Center for Electronics and Microtechnology (CSEM), 7302 Landquart, Switzerland; hui.chai-gao@csem.ch (H.C.-G.); yevhen.shynkarenko@csem.ch (Y.S.); stefano.cattaneo@csem.ch (S.C.)

**Keywords:** biosensor, allergy, allergen, IgE, antibody, microfluidics, point of care, fluorescence, optical reader, portable reader

## Abstract

With the steady increase in allergy prevalence worldwide, there is a strong need for novel diagnostic tools for precise, fast, and less invasive testing methods. Herein, a miniatured fluorescence-based biosensing system is developed for the rapid and quantitative detection of allergen-specific immunoglobulin-E. An antibody-based fluorescence assay in a microfluidic-patterned slide, combined with a custom-made portable fluorescence reader for image acquisition and user-friendly software for the data analysis, enables obtaining results for multiple allergens in just ~1 h with only 80 μL of blood serum. The multiplexed detection of common birch, timothy grass, cat epithelia, house dust mite, and dog epithelia shows quantitative IgE-mediated allergic responses to specific allergens in control serum samples with known total IgE concentration. The responses are verified with different control tests and measurements with a commercial fluorescence reader. These results open the door to point-of-care allergy screening for early diagnosis and broader access and for large-scale research in allergies.

## 1. Introduction

The incidence of immune-mediated disorders such as allergies is on a consistent rise [1,2]. Yet, there remains a significant gap in our understanding regarding the interplay of various factors such as genetic predisposition, exposures during prenatal and postnatal life, and environmental influences in the initiation and progression of these conditions [3].

The current landscape of allergy diagnostics, while rich with historical significance and clinical utility, highlights a pressing need for innovation to enhance patient comfort and provide quantitative data for large-scale research and diagnostics [4]. Traditional methods such as the Skin Prick Test (SPT) and oral food challenges, although foundational in the identification and confirmation of allergen sensitivities, are often criticized for their invasiveness and the discomfort they may cause to patients [4,5,6]. Also, these techniques face limitations in their qualitative nature, which may not fully satisfy the requirements of modern, data-driven allergy research and personalized medicine approaches.

As the field progresses towards more sophisticated molecular diagnostics and precision treatments, there is a noticeable demand for developing less painful, more quantitatively precise methodologies [4,7]. This shift aims not only to improve patient experience but also to enable the detailed, large-scale data collection necessary for advancing our understanding of allergic diseases [8]. Innovative solutions are urgently needed to bridge the gap between traditional practices and the potential for targeted, data-rich diagnostic approaches, thus opening the door to more effective and less invasive detection [9,10,11]. Other methods of significant importance for allergy diagnostics is the measurement of specific immunoglobulin-E (sIgE) in serum by enzyme-linked immunosorbent assay (ELISA) and fluorescence enzyme immune assay (FEIA) [7]. While fluorescence-based assays are generally analyzed in standard laboratory settings, after the shipment of the blood samples, and using commercial microarray scanners, the long time needed for obtaining the results, their cost, and the size of the commercial scanners render these methods impractical for point-of-care settings, limiting broader and faster access to allergy diagnostic tools.

In this work, we present the development of a novel portable multiarray system tailored for specific allergen profiling. The multiarray system is engineered on microfluidic chips sized equivalently to standard microscopy slides and is integrated within a microfluidic channel featuring functionalized microstructures with recombinant proteins of allergens. To ensure the reliable detection of sIgE, an automated sample-on-chip processing system has been utilized, employing fluorescence-labeled antibodies to detect the signal from each pillar with different allergens. Moreover, to facilitate simple measurements of fluorescence signals and automated quantification of the sIgE responses, a compact, cost-effective, and rapid fluorescence reader has been developed. This reader is equipped with cross-platform software having a user-friendly interface, further enhancing its accessibility and utility.

This platform concept holds promise in facilitating rapid screening of allergen specific IgEs in various settings, such as doctor’s offices, pharmacies, or university laboratories, for studying allergic disease causation and possible prevention strategies.

## 2. Materials and Methods

The microfluidic system, resembling a standard microscopy slide, was fabricated through injection molding of polycarbonate, with pillars of 300 μm in diameter and spaced 500 μm center to center in the same channel, with a channel width of 750 μm. The slides underwent coating with a dextran-based photo-linker polymer (OptoDex^®^, custom from CSEM, Landquart, Switzerland), serving for immobilizing allergens onto the micropillars, passivating the surface to mitigate non-specific bindings, and rendering the microfluidic channel hydrophilic.

Allergen recombinant proteins (including cat and dog epithelia, house dust mite, and common birch) and one allergen extract (timothy grass) were precisely dispensed onto the slides using a high-precision dispenser (sciDROP PICO, Scienion AG, Berlin, Germany) adding ~400 pL drop per pillar of protein solution, at an optimized concentration of 0.8 mg/mL in all cases, each allergen deposited six times for statistical analysis. All the recombinant proteins were purchased from Inbio (Cardiff, UK) and the allergen extract from BÜHLMANN Laboratories AG (Schönenbuch, Switzerland). Betula pendula (rBet v 1) was purified from Pichia pastoris culture by HPLC gel filtration; Felis domesticus (rFel d 1) from Pichia pastoris, clone N103Q, N-glycosylation site N103 mutated, was purified by multi-step column chromatography; house dust mite (rDer p 1) from Pichia pastoris (clone N52Q, N-glycosylation site N52 mutated) was purified by affinity chromatography; Canis familiar (rCan f 1) from Pichia culture supernatant using His-Trap chromatography. All recombinant proteins had a purity > 95% by SDS-PAGE. Finally, Timothy grass (BAG-G6) was an allergen extract (natural allergen) from Phleum Pratense. Also, Atto 647 Bioreagent (Sigma Aldrich, Buchs, Switzerland), was used to label BSA (Bovine serum albumin) for the fluorescence signal calibration, with concentrations from 10 to 5000 dye molecules/μm^2^, each concentration deposited in triplicate for each slide.

For the control tests in each slide, human IgE (Abcam, Amsterdam, The Netherlands) and human IgG (Jackson ImmunoResearch Labs, West Grove, PA, USA), at a concentration IgE/BSA and IgG/BSA of 0.4/0.2 mg/mL, were added into the slide, each solution deposited in six pillars.

Subsequently, a photo-immobilization process was initiated using a UV chamber (2 min at 20 mW/cm^2^, Beltron GmbH, Rödermark, Germany). With the process above, the slides with the immobilized proteins have long-term stability for at least 12 months when stored at 4 °C under vacuumed package (400 mbar nitrogen). Following lamination of a thin plastic foil (Simport T329-1, Simport-Saint-Mathieu-de-Beloeil, QC, Canada), control serum samples (PathTROL, TECOmedical AG, Sissach, Switzerland) were added into the fluidic slide channels to validate the efficacy of the multiarray. The control serum sample had a known total IgE concentration with verified presence of IgEs with specificity to the following allergens: timothy grass, cultivated rye (*S. cereale*), mugwort (*A. vulgaris*), house dust mite (*D. pteronyssinus*), house dust mite (*D. farinae*), cat epithelia, hazelnut, peanut, and milk.

Parallelized sample handling, accommodating up to 6 slides simultaneously, was achieved utilizing custom technology, with 80 μL of serum sample injected per slide with a peristaltic pump and incubated for ~30 min at 37 deg. Subsequent to sample deposition, detection antibodies (Mouse anti-hIgE) and AlexaFluor 647 Goat-Anti-mIgG (80 μL each, both from Life Technologies Europe, Zug, Switzerland) were introduced into the system and incubated for another ~45 min at 37 deg.

Measurement assessments were conducted utilizing both a commercial microarray reader (InnoScan 710, Innopsys, Carbonne, France) and a custom-made portable reader for a comparative analysis of their performance.

## 3. Results

### 3.1. Development of the Portable Fluorescence System

#### 3.1.1. Microarray Assay

**Figure 1:**
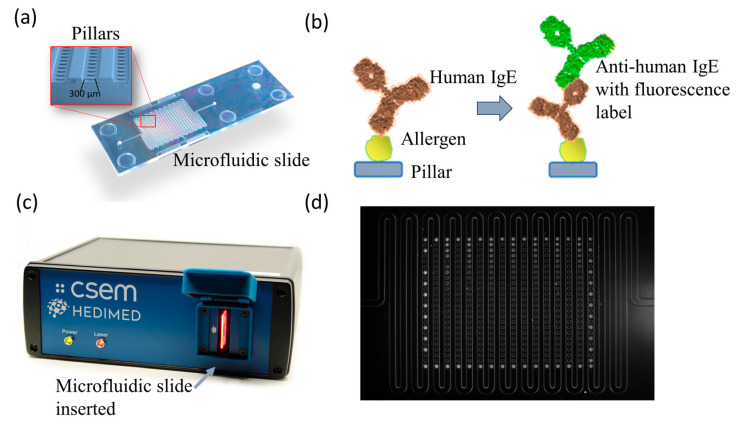
Portable system for allergen screening. (**a**) Image showing the microfluidic slide with a zoom on the micropillars; (**b**) simplified schematic of the biosensing assay on the slide; (**c**) image of the portable reader with a slide inserted; (**d**) the fluorescent signals on the micropillars with an image acquired with the portable reader and custom software.

A microarray chip featuring over 300 micropillars was designed (Figure 1a). This chip comprises a meandering channel featuring an array of microstructures functionalized with allergen recombinant proteins, with immobilization facilitated through the Optodex chemistry. Upon introduction of patient serum onto the functionalized chip, there is a recognition between the sIgEs and the allergen forming a complex, and the subsequent reaction with fluorescence labeled anti-IgE antibodies reveals sIgEs through distinctive fluorescence responses from each micropillar (Figure 1b).

#### 3.1.2. Portable Reader

**Figure 2:**
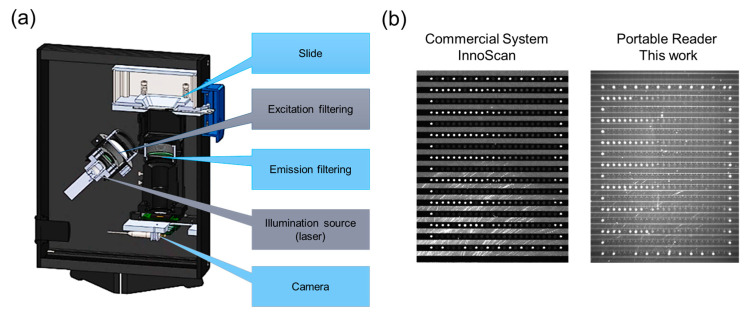
Portable reader components and comparison. (**a**) Schematic of the different components inside the developed portable reader; (**b**) Comparison of the data acquired by InnoScan and the portable reader, analyzing the microfluidic slide with different gradients of the fluorescent dye molecules Atto-BSA. Lower part shows correspondent line profile of the gradient from the lowest to the higher (24 points from about 10 to 5000 dye mol/μm^2^).

A portable reader was engineered for the fluorescence-based bio-detection (Figure 1c). Differing from commercial slide scanners that employ confocal scanning, this reader leverages fluorescence imaging, thereby reducing both size and cost while enhancing speed, albeit with a slightly reduced sensitivity (Figure 2). Key components of the reader include a camera, collection and beam-forming optics, a light source, emission and excitation filters, and a light trap (Figure 2a). A 642 nm fiber laser was selected as the light source, aligning with the spectral properties of the chosen dye Alexa-647. Light-forming optics ensure uniform sample illumination, while spectral filtering is accomplished through a combination of color glass and interferometric filters. Fluorescence emission was captured using a monochromatic CMOS camera featuring manual control for optimal imaging parameters. Notably, the portable reader boasts compact dimensions of 20 × 16 × 7 cm^3^, weighing a mere 1.5 kg, and comes at a fraction of the cost compared to conventional microarray readers, while still meeting the rigorous performance criteria essential for this assay.

Figure 2b shows the same slide read with the commercial InnoScan system and with the custom reader and correspondent line profiles. All 24 printed gradient points can be resolved with an InnoScan reader with 13 dye mol/μm^2^ distinguishable and with a standard deviation of the background signal equal to 6.4 fluorescence intensity (*n* = 124 points) and a sensitivity of 0.84 ± 0.04 fluorescence intensity signal/(dye mol/μm^2^), *n* = 5, giving a detection limit (LOD) calculated as 3σ/S (σ = standard deviation of the blank/S = sensitivity) equal to ~20 dye mol/μm^2^. In our compact reader, with a higher background level and higher noise, only 15 out of 24 concentration points can be differentiated from the noise, reaching the lowest distinguishable point of 96 dye mol/μm^2^, and with a blank signal variation equivalent to 76 fluorescence intensity (*n* = 124 points). Taking this into account, it can be calculated that the LOD of the portable device is 3σ/S, with S equal to 1.1 ± 0.06 fluorescence intensity signal/(dye mol/μm^2^), *n* = 5, so we have an LOD = ~200 dye mol/μm^2^ (σ = standard deviation/S = sensitivity), hence 10X higher than the commercial system.

The measurement spectral range can be defined by the set of filters chosen for the Alexa-647 dye and the camera quantum efficiency; for our chosen components, it is ~670–1100 nm. Considering the dynamic range based on the normalized dye molecule concentration characterization, the LOD was estimated to be 200 dye mol/μm^2^ as reported above, and the higher concentration could be measured by reducing the integration time and analog gain of the camera. So, theoretically, by using both factors, samples with dye concentration from ~200 dye mol/μm^2^ up to about ~10,000,000 dye mol/μm^2^ could be measured with the same system using only software control without any hardware changes. However, reaching a very high dye concentration does not make practical sense, and the current upper limit of the range used in the slides is 5000 dye mol/μm^2^, which is still in the linear range. Then, the signal-to-noise level for the single measurement without variation in the integration time is estimated for a relevant signal level to be about SNR = ~130 (Signal/NoiseRMS = 10,000/76).

#### 3.1.3. The Custom Software

**Figure 3:**
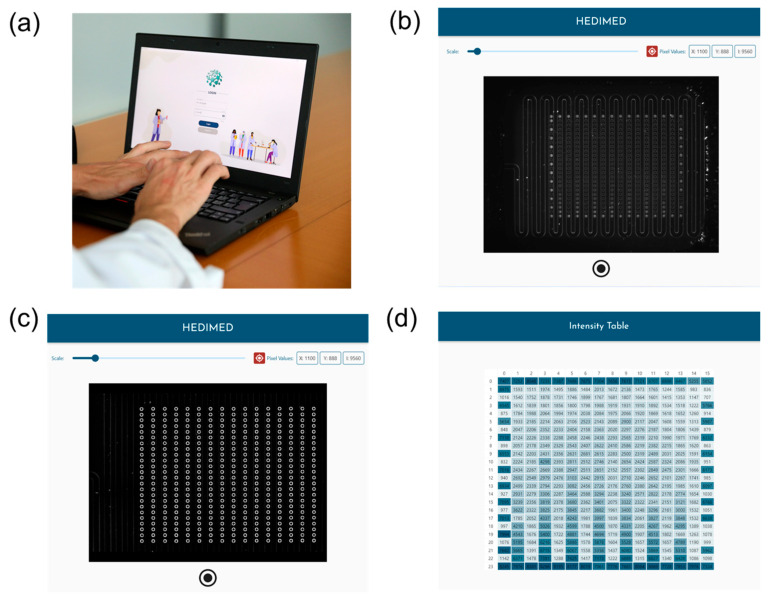
Images showing the custom software features. (**a**) Portable reader connected to the laptop with the program and login into the user interface; (**b**) the feature inside the program to acquire the image of the slide; (**c**) automated pillar recognition; (**d**) automated fluorescence data extraction and possibility to export in different Excel reports; the colors represent different fluorescence intensities.

The portable reader allows for full-slide imaging, markedly reducing readout times, as shown in Figure 1d and Figure 2b with the pictures acquired from the portable reader with the custom software. The software has a locally hosted backend written in Python code, which is responsible for all the computational parts and data management. Also, we have developed a user-friendly frontend which is programmed using the software Flutter (Figure 3).

Inside the software, you can login to your user profile (Figure 3a), acquire in about 1 s the image of the slide (Figure 3b), then automatically detect and identify the pillars in the slide (Figure 3c), and visualize and process the fluorescence intensity data extracted from the image in a table format (Figure 3d). Subsequently, the data can be exported and analyzed identically to the data extracted from commercial devices in a customizable excel sheet showing several resulting fluorescence intensity bar plots, as the ones reported in the data in the next paragraph. The fluorescence frame with high concentration of Atto/BSA dye enables the automated recognition of the area with the pillars.

The imagining area was optimized for our microfluidic slide to be about 2 cm × 1.5 cm. As for image acquisition speed, we acquire 10 images (~1 s); then, we take the averages of the images to reduce noise (basically instant, ~3 ms), and finally, we have the localization of points (~1 s) resulting in a total time of ~2 s. Considering the final software settings without any background subtraction and the rows of the microfluidic array from 1 to 8, the uniformity of the acquired signal can be estimated as the minimum background (I_min_) intensity divided by the average value (I_mean_), obtaining a percentage uniformity of (I_min_)/(I_mean_) = (1735/2317) × 100 = 75% (*n* = 61 for the mean). Considering the frame around the whole slide, we noticed a decrease in uniformity (65%, *n* = 76) possibly due to a non-uniform illumination, which could be corrected by hardware modification, software correction, or in the assay by performing a calibration with the dye molecule in both sides of the slide. In the reported data below, the results are all for allergens and calibration dye deposited in the first eight rows of the microfluidic array, hence with the 75% estimated uniformity.

### 3.2. Specific IgE Detection in Control Serum Samples

**Figure 4:**
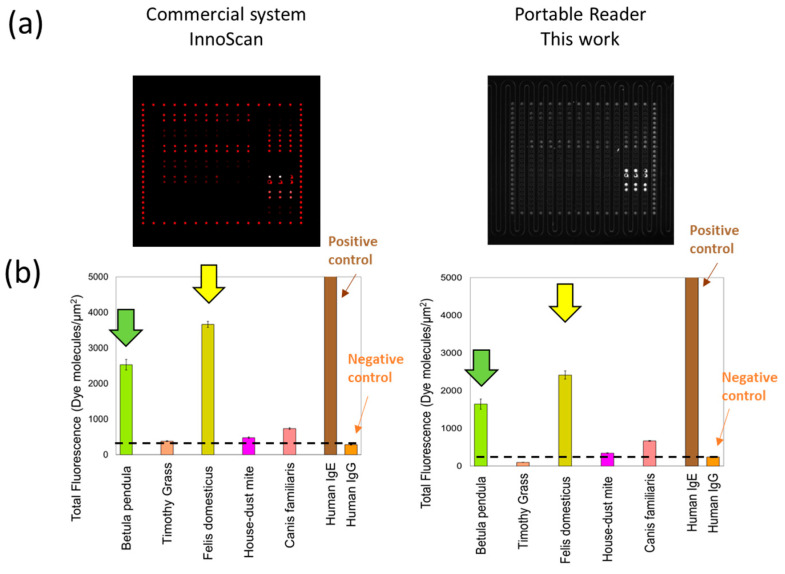
Results for specific IgE detection. (**a**) Optical image acquired by InnoScan and the portable reader, using a microfluidic slide with different recombinant allergens; (**b**) Relative fluorescence signals extracted and converted in dye molecules/μm^2^ based on the internal Atto BSA dye calibration. Tested with control serum (274 IU/mL total IgE). The dashed lines indicate the signal over which there is a significant IgE signal, highlighting the maximum cross-sensitivity signal from the IgG negative control.

The performance of the portable reader was evaluated through comparison with the commercial confocal microarray reader after the allergen immobilization and incubation with control serum. Control serum samples with known total IgE concentration, equivalent to the minimum IgE concentration produced in case of the possible presence of an allergic condition such as allergic rhinitis, asthma, and atopic dermatitis [12,13], are used for the validation of our assay and reader. The images acquired with both readers can be seen in Figure 4a. The fluorescence signal obtained from the microarray system is depicted in Figure 4b. Utilizing human IgE positive control signals (represented by the brown bar) along with a negative control (human IgG, depicted by orange bar), the crosstalk threshold can be established. All the reported signals are <5 to 10% of variations between the six replicated with the same allergen in different pillars and the control signals in triplicates.

The results demonstrate the detection capability for different allergens, particularly common birch (Betula pendula, indicated by green bars) and cat epithelia (Felis domesticus, depicted by yellow bars) with both the readout systems. The signal for house dust mite (Der. pteronyssinus) and dog epithelia (Canis familiaris), while surpassing the IgE crosstalk signal, has a much lower signal indicating low specific IgEs present for these allergens in the assay. Specifically, for the commercial reader, the crosstalk signal for the commercial scanner is equal to 281 ± 12 dye mol/μm^2^, and the signal for house-dust mite and Canis familiaris is 1.7× and 2.6× higher, respectively, and 9× and 13× higher for Betula pendula and Felis domesticus, respectively. In the case of the portable reader, the crosstalk signal extracted is of 239 ± 14 dye mol/μm^2^, being similarly slightly higher for house-dust mite and Canis familiaris (1.4× and 2.8×, respectively) and significantly higher for Betula pendula and Felis domesticus (6.9× and 10.1×, respectively). This corroborates with control serum samples testing positive for cat and house dust mites, while common birch and Canis familiars, not included in the manufacturer’s test, could not be directly confirmed. The reference test from the manufacturer can be requested online for the product PathTROL Allergy Control Sera-Level 2 (PW80201). It is possible that the very high specific IgE measured for common birch is due to a cross-reactivity with the specific IgEs for Cultivated rye [14], known to be present in the serum, and being in a similar birch and grass pollen family. The same is applied to different animal allergen, as also to Felis domesticus and Canis familiaris.

It is also important to remark that since there are appreciable total IgE levels in patients with allergen sensitization as well as those without, sIgE instead of total IgEs are the preferred method for determining the presence of allergic diseases [13]. Total IgE concentration is the addition of all the sIgE to the different allergens the individual has been exposed to; in non-allergic subjects, sIgE levels are very low and undetectable [7]. Thus, to identify the triggering antigen of allergic manifestations, one of the most common laboratory test requirements is the determination of sIgE concentration in serum, that can be performed in a point-of-care fashion with our device.

## 4. Discussion

The development of the portable fluorescence reader presents a viable option for point-of-care allergy diagnostics and broader biosensing applications. Its compact design (size 20× smaller than standard commercial readers), lower cost (40× lower considering the prices of the current components used, ~1.5 kEUR), and rapid readout time (60× faster considering 1 min scan with Innoscan versus 1 s with the portable reader) make it highly accessible, with features including fast image acquisition and automated result computation enhancing usability. The signal uniformity in the whole slide can be easily improved by software correction or minor hardware and assay modification. The portable solution demonstrated significant IgE-sensitivity for specific allergen detection, with a performance comparable to the commercial system in terms of increased specific IgE signal over the IgG negative control signal. The system’s adaptability to other fluorophores further underscores its versatility and potential for multi-allergen, multi-antibody, and multi-analyte detection in diverse clinical settings. The system also facilitates the parallel testing of up to 88 different allergens, considering the current protocol and number of replicates for control and internal calibration, thus presenting novel avenues for allergen screening directly within clinical settings. The commercial product ImmunoCAP Immuno Solid-Phase Allergen Chip (ISAC) by Thermo Fisher Scientific, after further development through the European Union (EU) project MeDALL, was reported to include, in 2014, a total of 176 allergens, of which 127 were recombinant allergens and the rest natural allergens, [15,16] including multiple allergens from different allergen sources such as latex, peanut, wheat, alder, olive, Plane Tree, Goosefoot, annual mercury, and many others. Similarly, these different allergens can be deposited in our developed microfluidic slide and used for the parallel multiplexed analysis. Finally, we would like to point out that as a secondary antibody for the immunodetection, the commercially available fluorescent-labeled anti-human IgE antibody is still limited by its low activity. The development of a high-active fluorescent-labeled anti-human IgE antibody conjugate could be carried out in the future with possible collaborations. Thus, the sensitivity of the assay can be also increased by using labeled anti-human IgE antibody directly instead of the mouse anti-human IgE/anti-mouse IgG system currently employed. Further tests with several serum samples and standardization of the sIgE signal output in µg, ng, or IU through collaboration with allergen diagnostic associations and doctors could enable the widespread use of this technology in clinical settings and for standardized research.

## Data Availability

The data that support the findings of this study are available upon reasonable request.
